# Physiology: A Manual for Students and Practitioners

**Published:** 1893-03

**Authors:** 


Physiology: A Manual for Students and Practitioners. By Frederick A. Manning, M.D., Attending Surgeon, Manhattan Hospital, New York. Being Volume II. of the Students' Quiz Series, edited by Bern B. Gallaudet, M.D., New York. Published by Lea Brothers & Co., Philadelphia. Limp cloth, $1.00.

This well-condensed and systematic work is admirably adapted for students of physiology, and its plain, concise statements must make it useful and convenient to the practitioner. It is not intended to compete with nor to take the place of the more elaborate text-books, but to present the subject in such a manner as to fix in the memory facts already learned in less limited treatises.

The publishers present this series of manuals in an attractive, form, and the type used in this is more agreeable than in Volume I.

